# Predicting factors related with uncured hypertension after retroperitoneal laparoscopic adrenalectomy for unilateral primary aldosteronism

**DOI:** 10.1097/MD.0000000000016611

**Published:** 2019-07-26

**Authors:** WuYun BiLiGe, Chaoqi Wang, JiRiGaLa Bao, Dahai Yu, A Min, Zhi Hong, Xiangbao Chen, Min Wang, Dongmei Wang

**Affiliations:** a Department of Urology, Affiliated Hospital of Inner Mongolia University for the Nationlities; b Medical College of Inner Mongolia University for the Nationlities, Tongliao, P.R. China.

**Keywords:** adrenalectomy, predicting factor, primary aldosteronism, surgical outcomes, uncured hypertension

## Abstract

Although unilateral primary aldosteronism (PA) is the most common surgically correctable cause of hypertension, the cure rate varies widely. The predicting factors related to uncured hypertension are not completely established. This study was designed to determine predicting factors associated with resolution of hypertension after adrenalectomy for PA.

The records of unilateral PA patients who had undergone retroperitoneal laparoscopic adrenalectomy were retrospectively reviewed from January 2010 to December 2017 in a single center. Patient demographics and preoperative factors were analyzed, including age, sex, smoking history, family history of hypertension, the presence of diabetes, body mass index (BMI), systolic blood pressure, diastolic blood pressure, biochemical results and tumor characteristics. Univariate and multivariate Logistic regression analysis were used for statistical assessment.

126 patients with unilateral PA were enrolled, and the mean age at the time of surgery was 54.2 years. Of these patients, 74 (58.7%) were women, and the mean BMI and duration of hypertension were 26 kg/m^2^ and 61 months, respectively. Hypertension was cured in 46% patients, of the patients with uncured hypertension, 91% had improved control of hypertension. In univariate analysis, age (*P* = .03), BMI (*P* = .01), duration of hypertension >5 years (*P* = .03), preoperative antihypertensive agents>2 (*P* = .02), contralateral abnormalities (*P* = .03) were the main factors related to uncured hypertension after adrenalectomy. In multivariate regression analysis, uncured hypertension was independently associated with obesity (25.00–29.99: odds ratio [OR], 2.97, *P* < .02; ≥30: OR, 6.42, *P* < .01), duration of hypertension >5 years (OR, 6.25, *P* < .01), preoperative antihypertensive agents >2 (OR, 5.30, *P* < .001), and contralateral adrenal abnormalities (OR, 8.38, *P* < .01).

The hypertension cure rate of unilateral adrenalectomy in PA is not high. Obesity, duration of hypertension >5 years, preoperative antihypertensive agents >2 and contralateral adrenal abnormalities were independently associated with uncured hypertension.

## 1 Introduction

Primary aldosteronism (PA) was first described by J. Conn in 1955 and encompasses a group of disorders in which aldosterone production is inappropriately high for sodium status, relatively autonomous of the major regulators of secretion (angiotensin II, plasma potassium concentration), and is not suppressed by sodium loading.^[1,2]^ It causes hypertension, occasionally hypokalemia and alkalosis. Some studies have reported that the prevalence of PA is reported as 5% in the general hypertensive population, increasing to 10% in referred populations and 15–20% in patients with treatment-resistant hypertension.^[3–5]^

Primary aldosteronism leads to long-term fibrosis and remodeling in critical organs due to high aldosterone level and subsequent hypertension.^[6,7]^ Several studies have shown a higher prevalence of cardiovascular, cerebrovascular and renal morbidity, and mortality in patients with PA than in patients with primary hypertension matched for age, sex, and blood pressure.^[8–10]^ Therefore, PA could be considered a serious health issue. Adequate treatment of PA leads to significant reduction of morbidity and mortality through cure or improvement of aldosteronism and hypertension.^[11,12]^

Disease management depends on the subtype and patient characteristics. Primary aldosteronism is typically caused by an adrenal adenoma and unilateral or bilateral adrenal hyperplasia.^[13]^ Current guidelines recommend screening at-risk patients with the ratio of plasma aldosterone to plasma renin (aldosterone–renin ratio), and further subtype characterization of either an aldosterone-producing adenoma or adrenal hyperplasia is based on axial imaging and venous sampling as indicated.^[2,14]^ A benign aldosterone-producing adenoma and unilateral adrenal hyperplasia accounts for 75 to 80% cause of PA, and it can be cured by surgical intervention.^[2,15]^ In patients with idiopathic aldosteronism, the adrenal gland usually reveal bilateral hyperplasia, it should be medically treated with a mineralocorticoid receptor antagonist and some other drugs.^[16–18]^

Although unilateral PA is the most common surgically correctable cause of hypertension, the rates of postoperative resolution of hypertension vary after unilateral adrenalectomy, despite normalization of the biochemical marker abnormalities. Systematic reviews and meta-analyses have indicated clinical cure (postoperative normotensive state without the use of antihypertensive medications) on pooled data in 42%, 50%, and 52% of patients.^[16,19,20]^ It is difficult to identify the subgroup of patients who would need no antihypertensive medication after adrenalectomy. Several studies have evaluated the related factors predicting postoperative resolution of hypertension, including sex, age, hypokalemia, duration of hypertension, number of antihypertensive drugs, and treatment before vascular remodeling.^[21,22]^ However, the risk factors vary widely between research centers. The aim of this study was to further investigate preoperative characteristics of patients undergoing retroperitoneal laparoscopic adrenalectomy for unilateral PA in an effort to identify risk factors associated with uncured hypertension.

## 2 Methods

The electronic patient database of the Affiliated Hospital of Inner Mongolia University for the Nationalities was queried for patients diagnosed with unilateral PA who had undergone retroperitoneal laparoscopic adrenalectomy from January 2010 to December 2017. All enrolled cases required a complete follow-up record. This study was approved by the Ethics Committee of the Affiliated Hospital of Inner Mongolia University for the Nationalities. Written informed consent from the patients was obtained.

Primary aldosteronism was diagnosed in accordance with the US Endocrine Society guideline.^[2]^ The criteria used to establish the diagnosis were a history of hypertension (with or without hypokalemia) with biochemical evidence of hyperaldosteronism and suppressed plasma renin activity. All patients with PA undergo adrenal computed tomography (CT) or magnetic resonance imaging (MRI) as in the initial study in subtype testing to exclude large masses that may represent adrenocortical carcinoma and to help make the distinction between unilateral and bilateral adrenal disease. Adrenal venous sampling (AVS) was used for lateralization for patients who had bilateral adrenal abnormalities according to the CT or MRI.

Patient demographics and preoperative factors analyzed included age, sex, smoking history, family history of hypertension, the presence of diabetes, body mass index (BMI), systolic blood pressure (SBP), diastolic blood pressure (DBP), biochemical results and tumor characteristics. Information regarding the duration of hypertension was also collected, as well as the number of antihypertensive agents at the time of operation. According to previous reports, the duration of hypertension was divided into two groups (≤5 years, >5 years), and the antihypertensive agents were divided into ≤2 and >2 groups. The principal outcome was cure of hypertension, which was defined as normal blood pressure (SBP < 140 mm Hg and DBP < 90 mm Hg) without aid of antihypertensive agents; The “uncured” patients were classified to note those patients who had normal BP receiving a lower or equal number of antihypertensive medications (improved group), and those who did not have a reduction in antihypertensives or remained hypertensive following surgery (not improved group).

### 2.1 Statistical methods

IBM SPSS Statistics version 22.0 was used for all statistical analysis. All quantitative normally distributed variables are reported as means with SDs. Categorical variables are presented as absolute numbers and percentages. Chi-square and Fisher exact tests were used for univariate analyses to determine the main risk factors related with uncured hypertension. Multivariate binary logistic regression was performed for further investigation if any parameter was found to be significant with univariate test. Odds ratios (ORs) were calculated, and OR >1 indicates an increased likelihood of uncured hypertension and an OR <1 indicates a decreased likelihood. In all analyses, two-sided hypothesis testing was carried out, and probability values < 0.05 were considered significant.

## 3 Results

From January 2010 to December 2017, 126 patients with unilateral PA had undergone a retroperitoneal laparoscopic adrenalectomy procedure. The electronic records of these patients were retrospectively assessed. All the procedures were successfully performed. The patient's characteristics are given in Table 1.

**Table 1:**
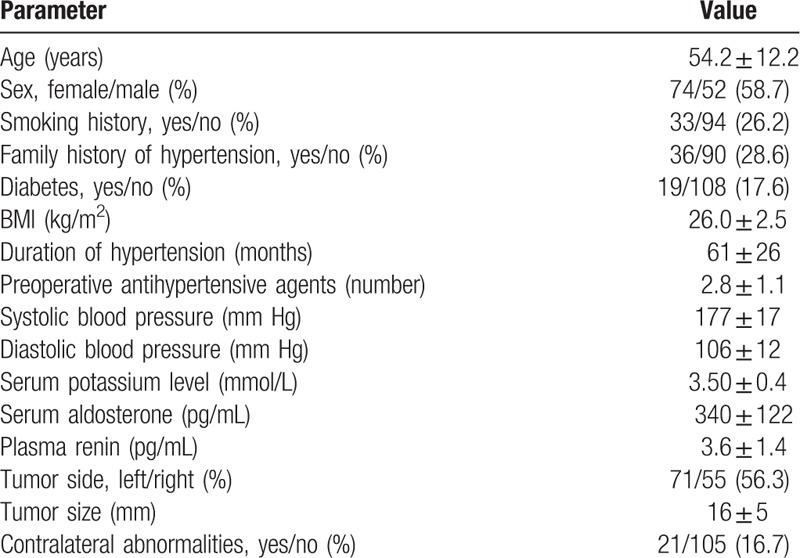
The patient's characteristics.

The median follow-up time was 42.2 months (ranging from 12 to 72 months). The results of biochemical outcome were normal in 118 patients, and hypertension was cured in 58 of 126 patients (46%). Of the patients with uncured hypertension, 62 of 68 (91%) had improved control of hypertension. All patients with hypokalemia had normal blood potassium levels after surgery.

In univariate analysis, age (*P* = .03), BMI (*P* = .01), duration of hypertension >5 years (*P* = .03), preoperative antihypertensive agents >2 (*P* = .02), contralateral abnormalities (*P* = .03) were the main factors related to uncured hypertension after retroperitoneal laparoscopic adrenalectomy for unilateral PA. However, sex, smoking history, family history of hypertension, diabetes, SBP, DBP, tumor side, and size were not significantly related (all *P > *.05). No significant differences were found in preoperative plasma potassium level, aldosterone and renin between the cured and uncured patients (all *P* *>* .05) (Table 2).

**Table 2:**
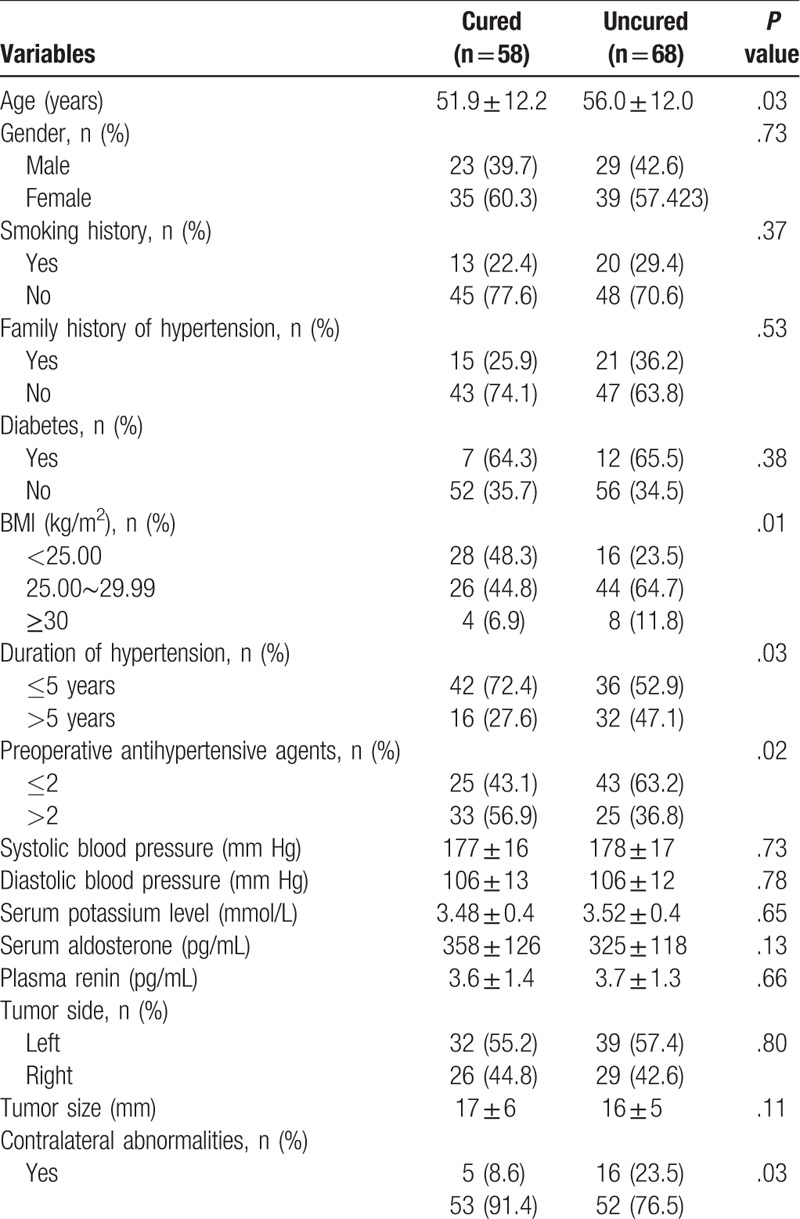
Univariate analysis of factors evaluated for an association with uncured hypertension.

In multivariate regression analysis, uncured hypertension was independently associated with obesity (25.00–29.99: OR, 2.97; *P* < .02; ≥30: OR, 6.42; *P* < .01), duration of hypertension >5 years (OR, 6.25; *P* < .01), preoperative antihypertensive agents >2 (OR, 5.30; *P* < .001), and contralateral adrenal abnormalities (OR, 8.38; *P* < .01) (Table 3).

**Table 3:**
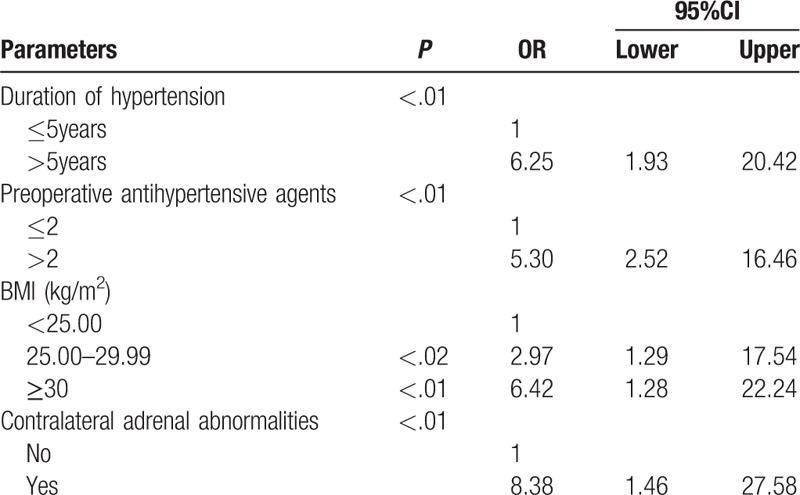
Predictive factors for uncured hypertension: outcome of multivariate binary logistic regression analysis.

## 4 Discussion

Aldosterone-producing adenoma and unilateral hyperplasia are curable by adrenalectomy. Since aldosterone-producing adenomas are usually smaller than 2 cm, laparoscopic rather than open, adrenalectomy is recommended.^[2,13]^ However, after unilateral adrenalectomy, not all PA patients are completely cured of their hypertension, despite normalization of the biochemical marker abnormalities. The results on clinical cures vary extensively across studies (22–84%).^[16,19,20,23]^ Uncured hypertension is most likely due to coexistent essential hypertension and subsequent irreversible vascular changes, induced by both hypertension itself and direct exposure to aldostrerone despite a metabolic cure.^[24,25]^ It has also been observed that the prevalence of hypertension in patients with uncured hypertension after adrenalectomy was almost the same as the prevalence of essential hypertension in a random population of the same age.^[26]^

It is particularly important to identify the factors predicting postoperative uncured hypertension, which could better inform the patient preoperatively. Several studies have evaluated factors predicting postoperative uncured hypertension, and scoring systems have been published with the intent to predict cure following adrenalectomy.^[21,27]^ However, no models have been developed to prioritize clinical improvement as an outcome measure.

In the present study, we found that age was related to uncured hypertension after adrenalectomy in univariate analysis. Some previous study also believed that age represented the main independent risk factors affecting hypertension cure rate.^[13,27]^ Aldosterone excess could cause vascular and perivascular inflammation, oxidative stress, and fibrosis. The incidence of left ventricular hypertrophy and myocardial infarction was reported to be higher in patients with primary aldosteronism than in those with essential hypertension. These damages of vascular and organs were irreversible, especially for elderly patients.^[24,28]^ It seemed that the older the patient is, the likelihood of such irreversible damage may occur, which may be the cause of the uncured hypertension. However, in our multivariate regression analysis, we did not find that age was independently associated with uncured hypertension.

Obesity is recognized as a risk factor for mortality, cardiovascular disease and metabolic disorders, such as hypertension, type 2 diabetes, and dyslipidemia.^[29]^ Many researches showed that obesity increases the chance of hypertension, and insulin sensitivity is significantly associated with hypertension.^[30,31]^ In our series, BMI was also predictive of uncured hypertension. Obesity is a known contributing factor to increased aldosterone production, and increased adiposity has also been linked to alterations in adrenal function, with increased circulating aldosterone levels.^[32]^ In turn, aldosterone exacerbates glucose intolerance through upregulation of the mineralocorticoid receptor.^[33,34]^ A recent study also show that BMI is an important contributing factor in resolving hypertension following adrenalectomy.^[35]^

The majority of our patients carried a hypertension diagnosis for more than 5 years. However, only 46% of our patients were cured, and 49.2% patients had improved in their hypertension. Interestingly, the duration of time in which they had been diagnosed with hypertension, was significantly longer in the uncured group, and duration of hypertension >5 years related with an uncured hypertension. Long-term hypertension causes atherosclerosis of small blood vessels and poor elasticity of blood vessels. Even if the aldosteronoma is removed, blood pressure will not return to normal.^[9,16]^ In addition, PA with concomitant essential hypertension has been suggested, with the majority of cases being secondary to essential hypertension.^[27]^ Therefore, early therapy with a receptor antagonist, early detection and removal of the aldosteronoma for PA patients may prove beneficial for blood pressure control, as well as limit blood vessels damage.

In our cohort, all patients received preoperative administration of the mineralocorticoid receptor antagonist, spironolactone. However, in addition to spironolactone, most patients required one or more other type of antihypertensive drugs. In multivariate regression analysis, uncured of hypertension was associated with the use of more than two antihypertensive agents. These variables probably reflect the fact that patients who need more preoperative antihypertensive agents may have concurrent refractory essential hypertension. One research recommend lifestyle interventions, including exercising, maintaining an ideal body weight, smoking cessation and minimal alcohol use, in patients who have an uncured hypertension after surgery.^[35]^

Due to the relatively high rate of discordance between AVS and imaging, AVS remains the gold standard and the most accurate way to differentiate between the unilateral and bilateral categories of PA.^[36,37]^ It plays a critical role in aiding the clinician to lateralize the aldosterone-producing adenomas. However, it is unclear in this subset of patients how the presence of a contralateral adrenal abnormality affects the results of adrenalectomy for PA.^[38]^ In our cohort, all of the patients who had a bilateral abnormalities were lateralized by AVS. However, contralateral abnormality was still a risk factor related to uncured hypertension. Such results may be related to the accuracy of AVS. AVS is more invasive testing and requires considerable technical skill, and its accuracy depends on a variety of factors.^[39]^ Dekkers et al designed a randomized control trial aimed at comparing CT- and AVS-based management in patients with confirmed PA. After a unilateral adrenalectomy, a total of 14 patients (15%), 9 in the CT subgroup (20%), and 5 (11%) in the AVS subgroup showed persistent PA. The authors concluded that AVS is an imperfect test to identify the aldosterone-producing adrenal abnormalities. Bilateral aldosterone-producing adenoma and hyperplasia can occur simultaneously,^[40]^ a standard and repeated AVS need to be given to these patients.

However, for the uncured patients in this cohort, most of them had improved blood pressure due to the removal of the cause of excess of aldosterone. The decrease of aldosterone level may determine an improvement in the cardiovascular and cerebrovascular outcome due to its pleiotropic effects.^[41–44]^ A meta-analysis suggested that the treatment of PA can induce regression of left ventricular hypertrophy, a surrogate for hard cardiovascular outcomes.^[45]^ Another recent research found that adrenalectomized aldosterone-producing adenoma patients are associated with lowers incident atrial fibrillation at long term.^[46]^ Therefore, early identification of PA patients who need adrenalectomy is a key measure to improve cardiovascular outcomes regardless of the decrease in blood pressure.

Our study has a few limitations. First, similar to almost all other studies regarding PA, the retrospective design may limit the generalizability of our results. The patients from a single center and small sample size could have recall and referral bias, mostly because of the low prevalence of PA, is one of the weaknesses of our study. In addition, the 24 h-ambulatory blood pressure monitoring was not done in all of the patients may be another limitation. Moreover, we had excluded the patients with incomplete follow-up record, which can also introduce selection bias.

## 5 Conclusions

The hypertension cure rate of unilateral adrenalectomy in PA is only approximately 50% of the patients in this study. Moreover, a large proportion of the patients with uncured hypertension after surgery may benefit from adrenalectomy in significant reduction of BP and antihypertensive use. Obesity, duration of hypertension >5 years, preoperative antihypertensive agents >2 and contralateral adrenal abnormalities were independently associated with uncured hypertension. Given the limitations of this study, large-sample clinical trials are required to verify the rationale of potential preoperative predictors in developing a new and effective prediction model.

## Acknowledgments

This study was supported by the Scientific Research Program of Inner Mongolia University for the Nationalities (No. NMDYB18080)

## Author contributions

Conceptualization: WuYunBiLiGe, JiRiGaLa, Dongmei Wang.

Data collection: Chaoqi Wang, Dahai Yu, Xiangbao Chen.

Formal analysis: Min Wang, Dongmei Wang.

Methodology: WuYunBiLiGe, Dongmei Wang.

Writing – original draft: WuYunBiLiGe, Chaoqi Wang, ZhiHong.

Writing – review & editing: AMin, Dongmei Wang, JiRiGaLa.

**Conceptualization:** BiLiGe WuYun, JiRiGaLa Bao, DongMei Wang.

**Data curation:** Chaoqi Wang, Dahai Yu, Xiangbao Chen.

**Formal analysis:** Min Wang, DongMei Wang.

**Methodology:** BiLiGe WuYun, DongMei Wang.

**Writing – original draft:** BiLiGe WuYun, Chaoqi Wang, Zhi Hong.

**Writing – review & editing:** JiRiGaLa Bao, A Min, DongMei Wang.
